# Editorial: Integrative techniques to alleviate abiotic stress in plants using plant growth promoting bacteria and fungi: mechanisms, interactions, and applications

**DOI:** 10.3389/fpls.2025.1686762

**Published:** 2025-09-09

**Authors:** Muhammad Naseem, Amjad Iqbal, Muhammad Qadir, Anwar Hussain

**Affiliations:** ^1^ College of Natural and Health Sciences, Department of Environmental Science and Sustainability, Zayed University, Abu Dhabi, United Arab Emirates; ^2^ Department of Bioinformatics, Biozentrum Am Hubland, University of Würzburg, Würzburg, Germany; ^3^ Department of Food Science and Technology, Abdul Wali Khan University Mardan, Mardan, Pakistan; ^4^ Hunan Key Laboratory of Plant Functional Genomics and Developmental Regulation, College of Biology, Hunan University, Changsha, China; ^5^ Department of Botany, Abdul Wali Khan University Mardan, Mardan, Khyber Pakhtunkhwa, Pakistan

**Keywords:** plant production, sustainable production, PGPMs (plant growth-promoting microorganisms), Arbuscolar mycorrhizal fungi, biofertilizers, food security

Agricultural sustainability is under unprecedented pressure. Rapid urbanization, shifting land-use patterns, and the expansion of non-grain production on fertile soils have reduced the land available for staple crops, challenging global food security (Liu et al., 2021; Zhang et al., 2023). This has pushed scientists and policymakers to seek biological solutions that can restore productivity, improve soil quality, and reduce dependence on chemical inputs (Singh et al., 2020). A growing body of research points toward plant growth-promoting microorganisms (PGPMs), a diverse group that includes bacteria, fungi, and mycorrhizae, as a powerful tool in meeting these challenges (Vessey, 2003; Bashan and de-Bashan, 2010). The main goal of the topic call was to assemble recent studies that focus on the strategic application of PGPMs as a means to enhance, or at a minimum preserve, crop productivity under abiotic stress conditions. PGPMs are recognized for their ability to improve plant resilience by modulating physiological responses, enhancing nutrient uptake, and inducing systemic resistance against pathogens and pests. Leveraging these mechanisms offers a sustainable approach to mitigate yield losses and sustain agricultural productivity in the face of abiotic challenges. Here, we provide an overview of these seven (**Table 1**) articles published under our proposed topic of “Integrative Techniques to Alleviate Abiotic Stress in Plants Using Plant Growth Promoting Bacteria and Fungi: Mechanisms, Interactions, and Applications.” These recent contributions (**Table 1**) to our topic offer compelling evidence of the impact PGPMs have on plant productivity.

**Table 1:** Overview of key contributions to the topic.

Inoculum used	Crop system	Key traits & mechanisms	Broader implications
1. Aspergillus tubingensis (TL-B31f) & Talaromyces veerkampii (FY-R41f)	Rice	Li et al.: Phosphate solubilization, siderophore production, IAA synthesis, microbial community restructuring	Restores productivity on non-grain converted land; improves nutrient availability and soil health
2. Brucella rhizosphaerae (BB-3) & Delftia lacustris (MB-7)	Tomato	Kundal et al.: Phosphate solubilization (up to 91.2%), nitrogen fixation, HCN & ammonia production, IAA synthesis, siderophore production, biofilm formation	Potent bioinoculants for vegetable crops; enhance yield & root architecture
3. PGPB consortia + reduced N	Purple cauliflower	Collado-Gonzalez et al.: Hormone production, nutrient mobilization, improved nutrient uptake under N limitation	Eco-friendly strategy for high-value crops; reduces fertilizer dependency
4. Phosphite (Phi) + ptxD system	Multiple crops (conceptual application)	Li et al.: Bio-stimulant, fungicide, herbicide; phosphorus source in ptxD-engineered plants	Dual nutrient & weed control; reduces eutrophication; potential for resistant-weed management
5. Bacillus megaterium GXU087	Soybean	Qui et al.: Phosphate solubilization, nitrogen fixation, exopolysaccharide production, biofilm formation, ILA secretion	First report of ILA-mediated nodulation in soybean; targeted biofertilizer for legumes
6. Stand-age effects on Lycium barbarum microbiota	Wolfberry (long-term monoculture)	He et el. Alters diversity, composition, network complexity of bacterial & fungal communities; Proteobacteria & Ascomycetes dominant	Guides management of perennial crops for soil health & yield sustainability
7. Tulasnella BJ1	Bletilla striata (orchid)	Li et al.: Produces pectinase, protease, IAA; inorganic P solubilization; root colonization	Accelerates propagation of endangered orchids; boosts seedling quality

“Integrative Techniques to Alleviate Abiotic Stress in Plants Using Plant Growth Promoting Bacteria and Fungi: Mechanisms, Interactions, and Applications”.

## Plant growth-promoting fungi in Zhejiang Province (rice)

Li et al. reported that in Zhejiang Province, the problem of non-grain land conversion has been tackled by exploring the potential of plant growth-promoting fungi (PGPF) to restore productivity. From 108 soil samples, researchers identified 15 fungal isolates capable of solubilizing phosphate (11.91–31.65 mm), producing siderophores (17.09–24.66 mm), and synthesizing indole-3-acetic acid (IAA) in concentrations ranging from 8.79 to 96.50 μg/mL. Two isolates stood out: TL-B31f, identified as *Aspergillus tubingensis*, and FY-R41f, identified as *Talaromyces veerkampii*.

In rice trials, these fungi increased plant height by up to 15.30%, root length by over 43%, and fresh biomass by nearly 90% compared to controls. They also enhanced dry weight and significantly raised soil available phosphate by over 40% after 55 days. High-throughput sequencing revealed that inoculation altered the microbial community composition, suggesting that these fungi act not only through direct nutrient provision, but also by reshaping the soil microbiome. Soil properties such as pH, organic matter, and total phosphorus emerged as key modulators of this microbial restructuring.

## *Brucella rhizosphaerae* and *Delftia lacustris* in tomato cultivation

Kaundal et al. investigated plant growth-promoting bacteria (PGPB) and identified two potent strains: BB-3 (*Brucella rhizosphaerae*) and MB-7 (*Delftia lacustris*). These isolates exhibited a broad spectrum of growth-promoting traits, including phosphate solubilization (up to 91.2% for MB-7), nitrogen fixation, hydrogen cyanide production, ammonia release, siderophore synthesis (86.2% for BB-3), and robust biofilm formation. All strains produced IAA, with MB-7 reaching 89.1 µg/mL.

When *D. lacustris* was applied to tomato plants, growth improvements were dramatic: plant height rose by nearly 50%, shoot fresh weight by 32%, and root length by 45%. Such multifaceted benefits stem from improved nutrient acquisition, suppression of soilborne pathogens, and enhanced root system development. This makes *D. lacustris* a strong candidate for integration into commercial tomato production, particularly in reduced-input systems.

## Reduced nitrogen fertilization and PGPB in purple cauliflower

Similarly, Collado-Gonzalez et al. investigated the efficient use of fertilizers in combination with PGPMs, observing that excessive nitrogen fertilizer use remains a major environmental concern (Fowler et al., 2013). A study on purple cauliflower explored whether PGPB could offset the yield penalties of reduced nitrogen application. Plants grown with just 30% of the standard nitrogen dose and inoculated with PGPB exhibited a 51% increase in leaf sugar content compared to the uninoculated control. Protein content rose by 16–33%, depending on nitrogen level, and essential minerals like potassium and iron increased by 26% and 34%, respectively, under limited nitrogen conditions.

These findings suggest a synergy between PGPB inoculation and moderate nitrogen reduction, offering an eco-friendly approach that sustains crop yield and nutritional quality while reducing environmental impacts from excess nitrogen runoff.

## Phosphite (Phi) as a fertilizer and biocontrol agent

A comprehensive review by Li et al. examined the current state of phosphite (Phi) applications. While microbial inoculants act through biological mechanisms, some chemical amendments can be reimagined to work in harmony with these biological systems. Phosphite (Phi), a reduced form of phosphate, is stable, highly soluble, and resistant to soil fixation. Although plants cannot metabolize Phi on their own, it becomes a viable phosphorus source when paired with organisms carrying the *ptxD* gene (Lopez-Arredondo and Herrera-Estrella, 2012).

In agriculture, Phi acts as a bio-stimulant, fungicide, and herbicide. It enhances plant growth, improves stress tolerance, boosts fruit quality, and suppresses pathogens. The *ptxD*/Phi system holds promise for a dual-function approach, providing phosphorus nutrition while selectively controlling weeds, thereby reducing the risk of herbicide resistance. Additionally, Phi use can help mitigate eutrophication, aligning with sustainable nutrient management goals.

## *Bacillus megaterium* GXU087 and soybean nodulation

Qiu et al. identified that *Bacillus megaterium* GXU087 offers another bacterial route to sustainable productivity. This strain exhibits phosphate solubilization, nitrogen fixation, exopolysaccharide production, and biofilm formation. Notably, it secretes indole-3-lactic acid (ILA) at 232.7 ng/mL, which was shown to significantly enhance soybean nodulation and growth.

Pot trials confirmed that ILA application increased both nodule number and biomass, but without stimulating rhizobia proliferation, suggesting a direct effect on plant physiology rather than microbial population size. This is the first documented case of *B. megaterium* producing ILA as a growth and nodulation promoter in soybeans, opening new avenues for legume-specific biofertilizers.

## Soil microbiota dynamics in long-term *Lycium barbarum* cultivation

In another study, He et al. substantiated that microbial health is as important as plant growth when considering long-term productivity. Research into the continuous monoculture of *Lycium barbarum* (wolfberry) revealed that stand age significantly shapes soil bacterial and fungal communities. Diversity metrics (Shannon and Chao1 indices) tended to rise and then fall over time. Proteobacteria and Ascomycetes remained dominant across all ages, but the community assembly processes varied: bacterial communities were shaped mostly by stochastic processes, while fungal communities oscillated between stochastic and deterministic assembly (Zhou et al., 2020).

The complexity and stability of microbial networks peaked in 10- and 15-year-old stands, but changes in soil physicochemical properties driven by plant age strongly influenced these dynamics. This study underscores the importance of managing crop stand age to maintain soil microbial balance and sustain yields.

## Tulasnella BJ1 and the germination of *Bletilla striata*

Finally, Li et al. demonstrated that in the context of endangered orchids, mycorrhizal fungi like Tulasnella BJ1 proved critical for seed germination and early growth. Isolated from Bletilla striata roots, BJ1 was confirmed as a new Tulasnella strain through multilocus phylogenetic analysis. It produces pectinase, protease, and IAA, and can solubilize inorganic phosphorus.

When used in symbiotic seed germination, BJ1 significantly accelerated development: after four weeks, 74.23% of seeds reached stage 5, compared to 50.43% in controls. Seed dimensions and biomass were 1.8–3.7 times greater with BJ1 inoculation. Supplementing with L-tryptophan further boosted IAA production and germination rates, demonstrating the potential for controlled mycorrhizal partnerships in conservation and commercial propagation.

## Conclusions and future directions

Together, these seven studies (**Table 1**) highlight a mosaic of strategies, from fungal biofertilizers and bacterial inoculants to novel phosphorus chemistry, that can be tailored to specific crops, environments, and management goals. Common themes emerge: nutrient mobilization (especially phosphorus), hormone-mediated growth stimulation, pathogen suppression, and beneficial restructuring of the soil microbiome.

Looking forward, integrating microbial solutions into mainstream agriculture will require a combination of field-scale trials, regulatory approval frameworks, and farmer adoption programs (Schreiter et al., 2014). There is also a need for multi-strain consortia research to explore synergistic effects (Bashan et al., 2014), as well as omics-based studies to track microbial dynamics and functional genes in real time. For specialized systems, like Phi-based fertilization, genetic engineering of target crops could unlock dual nutrient and weed control systems with reduced environmental footprints (Lopez-Arredondo et al., 2014).

With agriculture confronting growing challenges, such as land scarcity, nutrient depletion, climate change, and biodiversity loss, microbial allies offer a way to produce more with fewer resources, such as reducing chemical inputs, minimizing environmental harm, and easing pressure on fragile ecosystems. By integrating these innovations strategically, future farming systems can be both highly productive and environmentally regenerative, safeguarding food security while preserving the planet.
